# Quercetin Regulates Key Components of the Cellular Microenvironment during Early Hepatocarcinogenesis

**DOI:** 10.3390/antiox11020358

**Published:** 2022-02-11

**Authors:** Itayetzi Reyes-Avendaño, Edilburga Reyes-Jiménez, Karina González-García, Dulce Carolina Pérez-Figueroa, Rafael Baltiérrez-Hoyos, Gabriela Tapia-Pastrana, Xariss Miryam Sánchez-Chino, Saúl Villa-Treviño, Jaime Arellanes-Robledo, Verónica Rocío Vásquez-Garzón

**Affiliations:** 1 Laboratorio de Fibrosis y Cáncer, Facultad de Medicina y Cirugía, Universidad Autónoma Benito Juárez de Oaxaca, Oaxaca 68120, Mexico; itayetzi.reyes94@cecad-uabjo.mx (I.R.-A.); edilreyesjimnez@yahoo.com.mx (E.R.-J.); k.igg@cecad-uabjo.mx (K.G.-G.); 1401110005@univas.mx (D.C.P.-F.); 2 CONACYT-Facultad de Medicina y Cirugía, Universidad Autónoma Benito Juárez de Oaxaca, Oaxaca 68120, Mexico; rbaltierrezho@conacyt.mx; 3 Laboratorio en Investigación Biomédica, Hospital Regional de Alta Especialidad de Oaxaca, San Bartolo Coyotepec, Oaxaca 71256, Mexico; gtapia@hraeoaxaca.gob.mx; 4 Catedra-CONACYT, Departamento de Salud El Colegio de La Frontera Sur, Unidad Villahermosa, Tabasco 86280, Mexico; xsanchez@ecosur.mx; 5 Departamento de Biología Celular, Centro de Investigación y de Estudios Avanzados del Instituto Politécnico Nacional, Ciudad de México 07360, Mexico; svilla@cell.cinvestav.mx; 6 CONACYT-Instituto Nacional de Medicina Genómica, Ciudad de México 14610, Mexico

**Keywords:** HCC, tumor microenvironment, preneoplastic lesions, quercetin, ABCC3

## Abstract

Hepatocellular carcinoma (HCC) is a health problem worldwide due to its high mortality rate, and the tumor microenvironment (TME) plays a key role in the HCC progression. The current ineffective therapies to fight the disease still warrant the development of preventive strategies. Quercetin has been shown to have different antitumor activities; however, its effect on TME components in preneoplastic lesions has not been fully investigated yet. Here, we aimed to evaluate the effect of quercetin (10 mg/kg) on TME components during the early stages of HCC progression induced in the rat. Histopathological and immunohistochemical analyses showed that quercetin decreases the size of preneoplastic lesions, glycogen and collagen accumulation, the expression of cancer stem cells and myofibroblasts markers, and that of the transporter ATP binding cassette subfamily C member 3 (ABCC3), a marker of HCC progression and multi-drug resistance. Our results strongly suggest that quercetin has the capability to reduce key components of TME, as well as the expression of ABCC3. Thus, quercetin can be an alternative treatment for inhibiting the growth of early HCC tumors.

## 1. Introduction

Hepatocellular carcinoma (HCC) is the most common primary liver cancer, and is ranked as the third leading cause of cancer death worldwide [1]. Its development is promoted by different risk factors, such as aflatoxin exposure, excessive alcohol consumption, chronic infection by hepatitis B and C virus, metabolic syndrome, diabetes, obesity, and nonalcoholic fatty liver disease. Either alone or in combination, these factors cause chronic liver damages that induce genetic and epigenetic alterations and as a result, several components of the microenvironment are modified [2].

Hepatocarcinogenesis is a complex process involving at least three well-categorized stages, initiation, promotion, and progression [3]. The early HCC stages are characterized by the development of preneoplastic lesions promoted by chronic liver damage, which eventually progress to established tumors [4,5]. It has been described that the tumor microenvironment (TME) plays a key role during hepatocarcinogenesis. The TME encompasses both cellular and noncellular components. The cellular component includes heterogeneous populations, such as cancer and non-cancerous cells, fibroblasts, and endothelial and immune cells [6]. The noncellular component is made up by cytokines, growth factors, and extracellular matrix (ECM) proteins, such as collagen and fibronectin. Together, these components contribute to the tumor development [6,7].

An important subpopulation of cancer cells is that of cancer stem cells (CSCs), which is characterized by the expression of specific markers, including epithelial cell adhesion molecule (EpCAM) and oval cell (OV6) protein [8,9]. Interestingly, a close relationship among CSCs, TME, and tumor persistence and resistance to treatments has been demonstrated [10].

Deposition of ECM in TME is a histopathological characteristic of HCC and constitutes a powerful stimulus for both its establishment and progression [11,12]. Increased deposition of ECM components leading to fibrosis is mediated by the differentiation of fibroblasts into myofibroblasts. Moreover, it has been described that cancer-associated fibroblasts are key modulators of the HCC initiation, progression, and metastasis as they promote the proliferation and invasion of cancer cells [13,14]. In addition, ECM proteins exert mechanical signaling that contributes to invasion and metastasis [15]. This evidence highlights the relevance of TME components in the HCC progression. Thus, regulation of TME components can be a therapeutic strategy to prevent HCC progression. Several investigations have developed therapeutic agents against TME components, either to eliminate them, block their interactions, or suppress the stimuli promoting hepatocarcinogenesis, invasion, and metastasis [16].

Failures of current HCC treatments are mainly due to intra-tumor heterogeneity and drug resistance, which can be addressed by different mechanisms such as the overexpression of transporters that favor the efflux of drug from cells, thus preventing them from exerting their therapeutic effect [17]. Increased expression of the ATP binding cassette subfamily C member 3 (ABCC3) transporter has been reported after administrating the first-line chemotherapy [18]; moreover, this transporter has been proposed as a marker of HCC progression and drug resistance [19]. Therefore, the regulation of ABCC3 level is an attractive therapeutic option for inhibiting the early HCC stages.

Antioxidants have been widely used as anticancer agents and have been shown to reduce drug resistance in HCC experimental models [20]. Quercetin has shown to have antioxidant, anti-inflammatory, and anticancer properties, and its effect has been studied in both in vitro and in vivo HCC models. Quercetin has the capability to regulate processes involved in HCC progression, such as inflammation, fibrosis, migration, apoptosis, and angiogenesis [21], as well as in the involved signaling pathways [22]. Quercetin modulates oxidative stress, a process closely associated to cancer development, through ROS depletion and increase of antioxidant system activity [23]. In addition, it inhibits inflammatory enzymes, such as cyclooxygenases (COX) and lipoxygenases, and modulates the activation of nuclear factor kappa B (NFκ-B) [24]. Its antifibrotic effects have been associated to the regulation of TGF-β/SMADs signaling pathway [25], and its antiproliferative effects have been associated to the induction of cell cycle arrest, inhibition of cyclins expression such as cyclin D1, and induction of CDK inhibitors such as p21 and p27, and that of apoptosis [22,26,27]. Its anticancer effects have been also closely related to the inhibition of metabolic activity, as well as to the induction of cell death through both activation of caspases and inhibition of survival signals regulated by either PI3K/AKT/ERK or JACK2/STAT3 signaling pathways [28,29,30]. In this line, we have previously shown that quercetin regresses HCC preneoplastic lesions by reducing oxidative stress, decreasing lipid peroxidation, and increasing the levels of antioxidant enzymes and glutathione; additionally, quercetin induces apoptosis of preneoplastic cells by activating caspase 9 and 3 [31,32], which was associated to the regulation of epidermal growth factor receptor and STAT5-associated signaling [33]. However, the capability of quercetin to revert altered TME components in the early HCC stages remains to be elucidated.

In this work, we evaluate the effect of quercetin on some TME components in the early hepatocarcinogenesis stages in an HCC rat model. Through histopathological and immunohistochemical analyses, we demonstrate that the administration of quercetin decreases preneoplastic lesions, fibrogenesis, glycogen accumulation, the expression of CSCs and myofibroblasts markers, the development of drug resistance denoted by decreased ABCC3 expression, and, as a result, the HCC progression. Our results encourage the consumption of quercetin as a preventive strategy in HCC high-risk populations.

## 2. Materials and Methods

### 2.1. Ethical Statement

All the experiments were carried out in accordance with the ethical principles of animal experimentation and under technical specifications for production, care, and use of laboratory animals of the Center for Research and Advanced Studies of the National Polytechnic Institute (CINVESTAV-IPN), under the approved protocol 0168-15.

### 2.2. Animals

Sixteen male Fischer 344 rats, with 180–200 g body weight (BW), obtained from the Laboratory Animal Production and Experimentation Unit (UPEAL) of CINVESTAV-IPN were used. All animals were kept under standard laboratory conditions, fed ad libitum, and housed in a controlled environment of 12 h light/dark cycle and temperature in ventilated cages kept in bio-bubbles, and protected by a double HEPA filtration system.

### 2.3. Experimental Protocol

**Figure 1:**
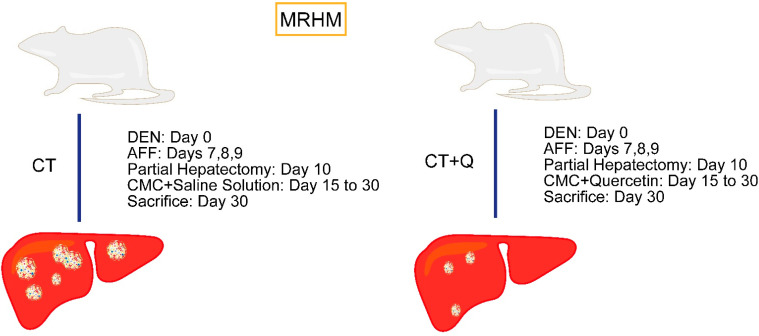
Induction of modified resistant hepatocyte model (MRHM) and quercetin treatment. Diethylnitrosamine (DEN) was administered as the initiating agent of carcinogenesis. Subsequently, 2-animofluorene (AFF) was administered as a promoting agent, and partial hepatectomy was performed as proliferative stimulus. Carcinogenic treatment (CT) group was also subjected to intragastric administration of carboxymethylcellulose (CMC). Carcinogenic treatment plus quercetin (CT + Q) group was subjected to quercetin 10 mg/kg BW. All animals were euthanized at day 30 post-carcinogenic treatment.

Rats were randomly divided into four experimental groups (4 rats per group). Two groups were subjected to MRHM for preneoplastic lesions induction as previously reported [33], and two other groups were used as control. For initiation, animals were intraperitoneally injected with a single dose of the carcinogen DEN (N0258, Sigma-Aldrich, St. Louis, MO, USA) at 200 mg/kg BW in saline solution. On days 7, 8, and 9, animals received an intragastric dose of 2-acetylaminofluorene (2-AAF; A7015, Sigma-Aldrich, St. Louis, MO, USA) at 20 mg/kg BW as promoting agent. On day 10, a 75% partial hepatectomy was performed as proliferative stimulus. From day 15 onwards, some animal groups were subjected to intragastrical administration of either quercetin (10 mg/kg BW; 32782, Sigma-Aldrich, St. Louis, MO, USA) or vehicle every other day and euthanized after eight administrations, as shown in Figure 1.

Negative control or non-treated group (NT) was only intraperitoneally injected with saline solution. The quercetin control group (NT + Q) was intraperitoneally administered with saline solution and quercetin was intragastrically administrated at 10 mg/kg BW in 0.5% carboxymethylcellulose (CMC); the selected dose of quercetin was based on previous reports [33]. For preneoplastic lesions induction, animals were subjected to carcinogenic treatment (CT) according to MRHM and were also intragastrically administrated with CMC. The last group was subjected to MRHM plus quercetin (CT + Q) and was also intragastrically administrated with quercetin at 10 mg/kg BW. Animals were euthanized under ether anesthesia on day 30. Livers were quickly recovered, washed in ice-cold sterile saline solution, fixed in 10% formaldehyde, dehydrated with standard alcohol-xylol procedure, and embedded in paraffin (Paraplast, Leica Biosystems, Buffalo Grove, IL, USA).

### 2.4. Histological Analysis

Slices 5 µm thick were cut and fixed on gelatinized slides. Liver histopathology was evaluated by hematoxylin and eosin (H&E) staining. Tissues were deparaffinized at 56 °C for two hours, rehydrated (xylol, xylol-ethanol, 96% ethanol, 90%, 80%, 70%, and finally tap water), immersed in hematoxylin of Harris (738, HYCEL, Jalisco, Mexico), acidic alcohol, ammonia solution, 96% ethanol, yellowish eosin (688, HYCEL, Jalisco, Mexico) and 96% ethanol; finally, tissues were dehydrated and mounted in synthetic resin.

Periodic acid Schiff (PAS) staining was performed to evaluate the liver glycogen content. After deparaffinization, tissues were immersed in periodic acid (P-7875, Sigma-Aldrich, St. Louis, MO, USA), washed, immersed in Schiff’s reagent (2919-125, HYCEL, Jalisco, Mexico), washed again, immersed in Harris’s hematoxylin, washed, dehydrated with standard alcohols-xylol procedure, and mounted in synthetic resin.

Masson’s trichrome (HT15, Sigma-Aldrich, St. Louis, MO, USA) staining was performed to evaluate collagen deposition. Tissues were deparaffinized, hydrated, and then immersed in a Bouin’s solution previously heated at 40 °C and washed. Then, tissues were stained with Weigert’s iron hematoxylin, washed, stained with Biebrich’s scarlet acid fuchsin, and washed again. Subsequently, tissues were treated with a solution of phosphotungstic acid and phosphomolybdic acid (1:1 volume/volume) and washed. Tissues were then stained in aniline blue, acetic acid, deionized water, 96% ethanol, xylol, and finally mounted in synthetic resin. After mounting, all tissues were observed under microscope (Primo Star, CARL ZEISS) at 10× and 40× magnification.

### 2.5. Immunohistochemical Analysis

Tissues were cut at 3 µm thickness and fixed on silanized slides. Immunohistochemical staining was performed for detecting OV6 (sc-101863, Santa Cruz Biotechnology, Santa Cruz, CA, USA), EpCAM (MA5-12604, ThermoFisher, Fremont, CA, USA), alpha-smooth muscle actin (α-SMA; A5228, Sigma-Aldrich, St. Louis, MO, USA), and ABCC3 (sc-59612, Santa Cruz Biotechnology, CA, USA) proteins. Tissues were deparaffinized and hydrated, and antigen retrieval was performed in citrate buffer pH 6. Tissues were stabilized with Tris-buffered saline (TBS) for EpCAM and OV6, and with phosphate-buffered saline (PBS) for α-SMA and ABCC3; subsequently, a solution of 0.1% TBS-Triton or 0.2% PBS-Triton, was added, respectively. Tissues sections were treated with 6% hydrogen peroxide in methanol and washed with either TBS or PBS, and tissues were blocked with 5% BSA. Then, primary antibodies were incubated overnight at 4 °C. Anti-mouse secondary antibody (A9044, Sigma-Aldrich, St. Louis, MO, USA) was incubated at 37 °C, and the signal was detected using DAB-PLUS substrate kit (00–2020, Life Technologies, Waltham, MA, USA). Tissue sections were counterstained with Harris’s hematoxylin, followed by acid alcohol, ammonia solution, dehydration, and finally, mounted in synthetic resin. Antibodies information are summarized in Supplementary Table S1.

### 2.6. Analysis of Photomicrographs

Sixty photomicrographs were taken at 10× and 40× magnification per animal, namely, 240 photomicrographs per immunohistochemical experiment. Preneoplastic areas were quantified by using photomicrographs of the entire liver tissue. Quantification of positive signal was performed using IMAGEJ^®^ v.2.3.0/1.53f software.

### 2.7. Statistical Analysis

Obtained data from images analyses were subjected to the Student’s *t*-test using the GraphPad Prism9 software (GraphPad, San Diego, CA, USA). All data are expressed as mean ± SD. *p* value < 0.05 was considered statistically significant.

## 3. Results

### 3.1. Quercetin Decreases Preneoplastic Lesions Area and Restores Liver Architecture

**Figure 2:**
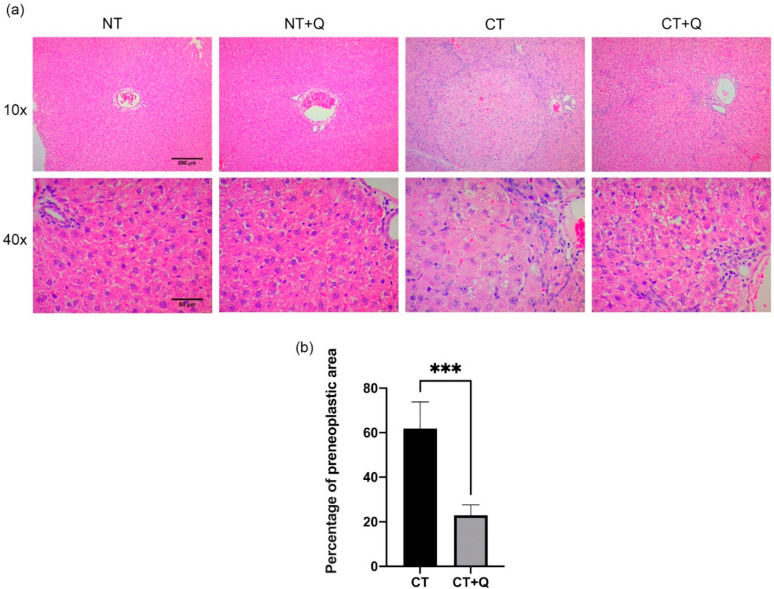
Effect of quercetin on preneoplastic lesions. (**a**) Representative H&E images of both control (NT, NT + Q) and MRHM (CT and CT + Q) liver tissues. Photomicrographs were taken at 10× and 40× magnification. (**b**) Preneoplastic lesions quantification of CT and CT + Q groups. Bars represent the mean ± SD, *p* < 0.001 (***). NT: untreated, NT + Q: untreated plus quercetin, CT: carcinogenic treatment, CT + Q: carcinogenic treatment plus quercetin.

First, the effect of quercetin on preneoplastic lesions was evaluated by H&E staining. While the liver architecture of negative controls (NT and NT + Q groups) was not modified, CT group showed preneoplastic lesions, hypertrophic hepatocytes containing macrovesicular vacuolar degeneration, preferentially, near the central vein, disarrangement of hepatocytes trabeculae and hepatic triads, presence of elongated cells, prominent nuclei, increased vascularization, and inflammatory infiltrate. Interestingly, CT + Q group decreased both the number and area of preneoplastic lesions, the inflammatory infiltrate, and restored the tissue architecture (Figure 2a). Quercetin decreased 62.89% (*p* < 0.001) the size of preneoplastic lesions in CT + Q group as compared with CT group (Figure 2b). This result strongly suggests that quercetin has the capability to inhibit the development of preneoplastic lesions and to restore the liver architecture alteration.

### 3.2. Quercetin Decreases Glycogen Accumulation within Preneoplastic Lesions and Promotes Its Redistribution

**Figure 3:**
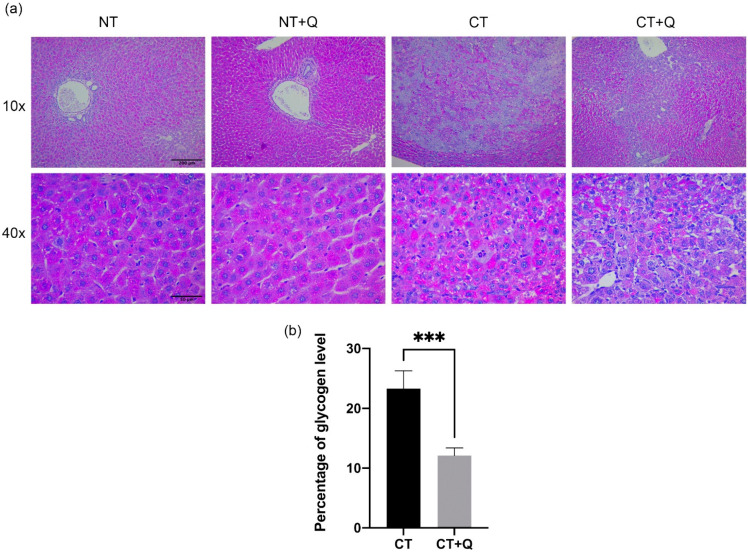
Effect of quercetin on glycogen accumulation. (**a**) Representative images of periodic acid Schiff (PAS) staining of both control (NT, NT + Q) and MRHM (CT and CT + Q) liver tissues. Glycogen-positive area is shown in deep pink. Photomicrographs were taken at 10× and 40× magnification. (**b**) Quantification of glycogen-positive area in CT and CT + Q groups. Bars represent the mean ± SD, *p* < 0.001 (***). NT: untreated, NT + Q: untreated plus quercetin, CT: carcinogenic treatment, CT + Q: carcinogenic treatment plus quercetin.

HCC is characterized by a deregulated metabolic activity, and it has been described that the accumulation of hepatic glycogen favors tumor development [34]. Therefore, we evaluated glycogen accumulation within preneoplastic lesions using PAS staining. While in NT group, glycogen was homogeneously distributed in the liver tissue, in NT + Q group a slight increase of glycogen was observed as compared with NT group. However, CT group increased the glycogen accumulation in central and peripheral areas of preneoplastic lesions. Of note, CT + Q group induced an evident diminution and redistribution of glycogen accumulation (Figure 3a). Glycogen-positive area decreased 48.02% (*p* < 0.001) in tissues of CT + Q group as compared to CT group (Figure 3b), indicating that quercetin decreases the accumulation of glycogen within preneoplastic lesions and favors its redistribution.

### 3.3. Quercetin Reverses Liver Fibrogenesis

**Figure 4:**
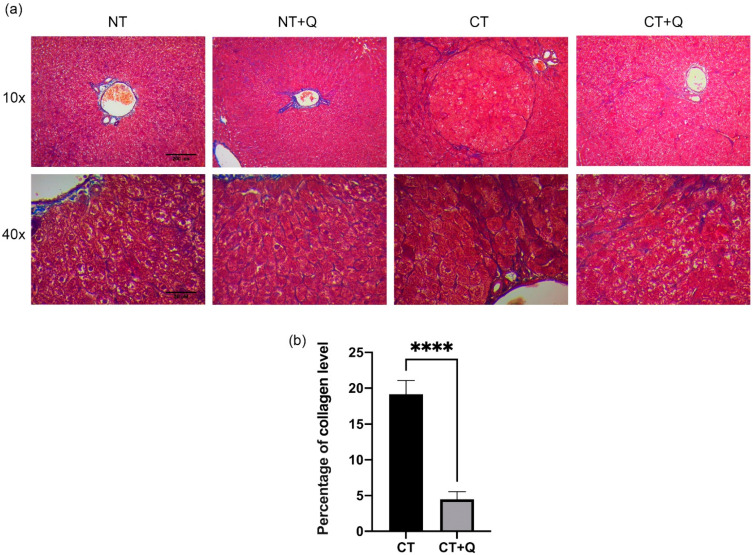
Effect of quercetin on collagen deposition. (**a**) Representative images of Masson’s trichrome staining of both control (NT, NT + Q) and MRHM (CT and CT + Q) liver tissues. Collagen-positive area is shown in blue. Photomicrographs were taken at 10× and 40× magnification. (**b**) Quantification of collagen-positive area in CT and CT + Q groups. Bars represent the mean ± SD, *p* < 0.0001 (****). NT: untreated, NT + Q: untreated plus quercetin, CT: carcinogenic treatment, CT+Q: carcinogenic treatment plus quercetin.

**Figure 5:**
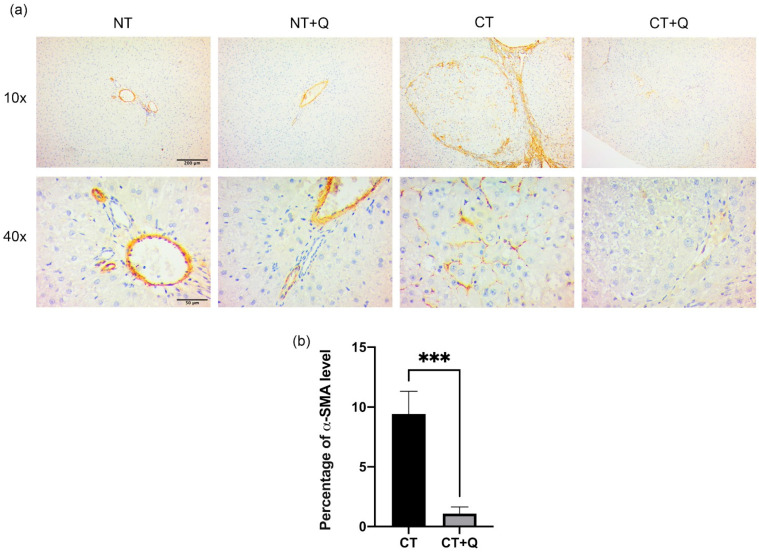
Effect of quercetin on marker of myofibroblasts. (**a**) Representative images of immunohistochemical staining for α-SMA detection in both control (NT, NT + Q) and MRHM (CT and CT + Q) liver tissues. Positive area is shown in brown gradients. Photomicrographs were taken at 10× and 40× magnification. (**b**) Quantification of α-SMA positive area in CT and CT + Q groups. Bars represent the mean ± SD, *p* < 0.001 (***). α-SMA: alpha-smooth muscle actin, NT: untreated, NT + Q: untreated plus quercetin, CT: carcinogenic treatment, CT + Q: carcinogenic treatment plus quercetin.

Fibrosis is a histopathological feature of HCC [11]; therefore, the effect of quercetin on collagen accumulation was evaluated by Masson’s trichrome staining. Basal collagen level was observed in NT and NT + Q groups (Figure 4a). While an evident increase in collagen deposition was induced by CT group, preferentially, in veins and hepatic triads, as well as in central and peripheral areas of preneoplastic lesions, quercetin decreased 76.54% (*p* < 0.0001) collagen deposition CT + Q group as compared with CT group (Figure 4b). Additionally, we also determine the presence of myofibroblasts, the main cells that produce ECM [35], by detecting the α-SMA marker through immunohistochemical staining. CT group increased the level of this marker by showing dense fibrotic areas, while CT + Q group decreased 88.51% (*p* < 0.001) α-SMA signal as compared with CT group (Figure 5a,b). This evidence suggests that quercetin reverses fibrogenesis induced during the early HCC stages, in part, by regulating collagen deposition and myofibroblast-dependent α-SMA levels.

### 3.4. Quercetin Decreases OV6- and EpCAM-Positive CSCs

**Figure 6:**
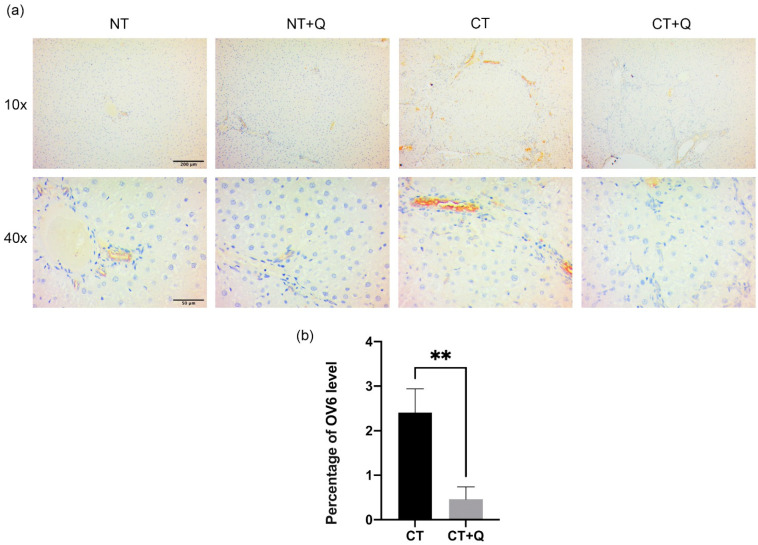
Effect of quercetin on OV6-positive CSCs population. (**a**) Representative images of immunohistochemical staining for OV6 in both control (NT, NT + Q) and MRHM (CT and CT + Q) liver tissues. Positive area is shown in brown gradients. Photomicrographs were taken at 10× and 40× magnification. (**b**) Quantification of OV6-positive area in CT and CT + Q groups. Bars represent the mean ± SD, *p* < 0.01 (**). OV6: oval cell, NT: untreated, NT + Q: untreated plus quercetin, CT: carcinogenic treatment, CT + Q: carcinogenic treatment plus quercetin.

**Figure 7:**
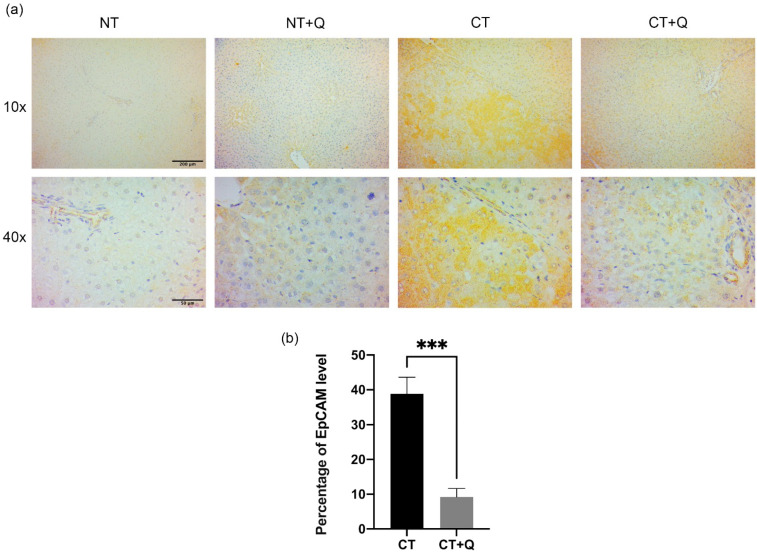
Effect of quercetin on EpCAM-positive CSCs population. (**a**) Representative images of immunohistochemical staining for EpCAM in both control (NT, NT + Q) and MRHM (CT and CT + Q) liver tissues. Positive area is shown in brown gradients. Photomicrographs were taken at 10× and 40× magnification. (**b**) Quantification of positive area for EpCAM in CT and CT + Q groups. Bars represent the mean ± SD, *p* < 0.001 (***). EpCAM: epithelial cell adhesion molecule, NT: untreated, NT + Q: untreated plus quercetin, CT: carcinogenic treatment, CT + Q: carcinogenic treatment plus quercetin.

The effect of quercetin on CSCs population was determined by detecting OV6 and EpCAM markers through immunohistochemical staining. Tissues from CT group showed increased expression of both markers, preferentially, surrounding preneoplastic lesions. Interestingly, the level of these markers decreased in CT + Q group as compared with CT group (Figure 6a and Figure 7a). OV6-positive area was reduced 73.53% (*p* < 0.01), and that of EpCAM was decreased 80.53% (*p* < 0.001) by quercetin (Figure 6b and Figure 7b). This result suggests that quercetin decreases OV6- and EpCAM-positive CSCs subpopulations during the early HCC stages.

### 3.5. Quercetin Decreases the Level of ABCC3 Prognostic Marker

**Figure 8:**
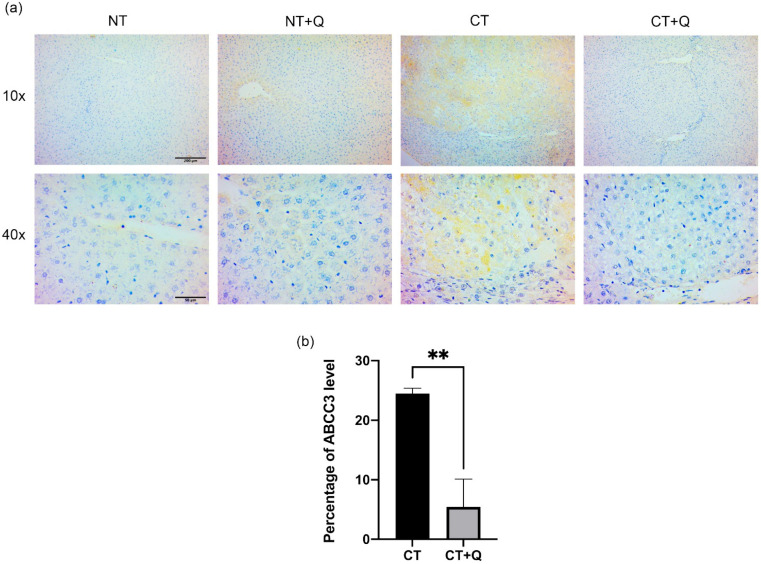
Effect of quercetin on ABCC3 level. (**a**) Representative images of immunohistochemical staining for ABCC3 in both control (NT, NT+Q) and MRHM (CT and CT+Q) liver tissues. Positive area is shown in brown gradients. Photomicrographs were taken at 10× and 40× magnification. (**b**) Quantification of ABCC3-positive area in CT and CT+Q groups. Bars represent the mean ± SD, *p* < 0.01 (**). ABCC3: ATP binding cassette subfamily C member 3, NT: untreated, NT + Q: untreated plus quercetin, CT: carcinogenic treatment, CT + Q: carcinogenic treatment plus quercetin.

The effect of quercetin on ABCC3 level was evaluated by immunohistochemical staining. Tissues from CT group showed increased levels of ABCC3, particularly, in remodeling preneoplastic lesions. Of note, tissues from CT + Q group showed a significant decrease of ABCC3 marker as compared with CT group. Furthermore, ABCC3 signal in tissues from CT + Q group was similar than that observed in NT and NT + Q groups (Figure 8a). ABCC3-positive area was reduced 77.82% (*p* < 0.01) in tissues from CT + Q group as compared with those from CT group (Figure 8b). This evidence suggests that quercetin has the capability to decrease the level of ABCC3, a drug resistance and prognostic marker of HCC.

## 4. Discussion

The high recurrence, lethality, and ineffective treatments have been the main challenges to fight HCC worldwide [1]. As TME plays a central role in the establishment and progression of HCC, its regulation has been attractive as therapeutic target. Quercetin, a flavonoid and powerful antioxidant, has been shown to have anti-proliferative, anti-angiogenic, and proapoptotic effects [21,36]. Previously, quercetin has been shown to reverse preneoplastic liver lesions in an HCC rat model [33]. Based on anticancer capabilities of quercetin, here we hypothesized that it can regulate components of TME during hepatocarcinogenesis.

Through histological analyses, we demonstrated that 10 mg/kg BW of quercetin significantly decreased the number and area of liver preneoplastic lesions by 62.89% in the rat, consistent with previous findings [33]. In liver cancer, the inflammation persistently promotes its development [37]. This process has been clearly observed by an inflammatory infiltrate through standard H&E staining. Our results show that the inflammatory infiltrate surrounding preneoplastic lesions was significantly reduced by quercetin. It has been reported that inflammatory factors contribute to the development of HCC tumor growth; interestingly, quercetin has shown the capability to decrease inflammation by inhibiting proinflammatory pathways, such as that of NF-κB and COX-2 in a human hepatoma cell line, and also by regulating PI3K/Akt and JNK activation [38,39]; these could be some of the possible molecular mechanisms by which quercetin inhibits the preneoplastic lesion growth.

As a chemoprotective agent, quercetin has been shown to suppress the activity of kinases involved in the growth and proliferation of cancer cells [40], and its anti-proliferative effects have been attributed to its capability to regulate metabolic reprogramming [41]. The antitumor effect of quercetin has been widely associated with its ability to induce apoptosis [42]. Quercetin can induce apoptosis by several mechanisms, including the regulation of the antioxidant system activity, as well as the suppression of p53 gene expression and that of BCL-2 protein level [43]. Additionally, the effect of quercetin on p53-independent cell death through the induction of both ROS and transcription factor EB has recently been studied through both the participation of Bid and the link between the induction of ferroptosis and apoptosis [44]. In addition, quercetin induces apoptosis through the Sestrin2/AMPK/mTOR pathway, which was associated with an increased intracellular ROS induced by quercetin, a mechanism independent of p53 participation [45]. It also regulates the proliferation of CSCs subpopulations and exerts antifibrotic effects by activating proapoptotic signaling [46]; thus, we argue that likely its capability to decrease the early hepatocarcinogenesis is closely associated to the activation of antiproliferative and/or proapoptotic mechanisms. In addition, other mechanisms are important, such as epithelial–mesenchymal transition and angiogenesis. The anti-angiogenic effect of quercetin has been determined in several experimental models. Quercetin has shown to either inactivate vascular endothelial growth factor (VEGF) or abrogate JAK2/STAT3 signaling pathway and as a result, the angiogenesis has been inhibited [30,46,47]. Additionally, the anti-angiogenic effect of quercetin has been closely linked to the decrease of VEGF-A, MMP2, and MMP9 expression [48], as well as to the inhibition of both tumor growth and angiogenesis by targeting VEGFR2 regulated by AKT/mTOR/P70S6K signaling pathway [49].

Tumor development involves metabolic reprogramming as a cell adaptive response to the TME [7]. Physiological and pathological stimuli can regulate glycogen metabolism in cancer cells. In this process, hypoxia, a key factor in carcinogenesis, upregulates enzymes that participate in glycogenesis [50]. High concentrations of glycogen have been described in several cancer cell lines, particularly in those undergoing neoplastic transformation [51]. Its accumulation in established clinical carcinomas promotes aggressive, and in hepatomas, it favors its maintenance in vitro [52,53]. Consistently, our results showed that in CT group, the highest glycogen accumulation was located within preneoplastic lesions. Interestingly, quercetin decreased glycogen accumulation and promoted its redistribution in the whole liver tissue; similar effects have been observed in a nonalcoholic liver disease model [54]. The mechanisms by which glycogen favors hepatocarcinogenesis have not fully clarified; however, it has been proposed that glycogen is the main source of energy in TME, as the result of an adaptive response to chemotherapy and radiotherapy [55]. In addition, the regulation of glycogen accumulation might inactivate signaling pathways involved in inflammation and hepatocarcinogenesis [56]; therefore, our finding strongly suggests that the redistribution of glycogen by quercetin may have influenced the reduction of preneoplastic lesions.

Liver fibrosis, which is characterized by aberrant accumulation of ECM components, constitutes a potent stimulus for the establishment of cancer [11]. Severity of fibrosis is directly correlated with the appearance of preneoplastic lesions and tumor establishment [57]. Fibrosis-promoted hepatocarcinogenesis is attributed to increased integrin-mediated signaling, generated by ECM hardening and effector cell migration [58,59]. Our results showed that quercetin reduces collagen deposition, a phenomenon also observed in models of carbon tetrachloride-induced liver fibrosis [60]. It has been reported that the antifibrotic effect of quercetin is mediated through the diminution of myofibroblast population [25]. Our results showed that quercetin diminished α-SMA level, a widely used myofibroblast marker. Several studies have suggested that quercetin modulates myofibroblast population by activating the intrinsic apoptosis pathway [61]. This evidence indicate that quercetin may induce myofibroblast apoptosis, and as a result, fibrogenesis was blocked. In has been reported that fibrogenesis induced by MRHM is largely mediated by DEN-induced ROS [62]. Interestingly, it has been reported that quercetin decreases ROS level [31]; therefore, its capability in reestablishing the antioxidant balance in cell microenvironment may be a mechanism that contributes to attenuating liver fibrosis during the early HCC stages.

TEM also includes CSCs populations which promote tumor heterogeneity, recurrence, metastasis, and drug resistance [63]. They can be identified by different markers, including OV6 and EpCAM, among others [8,9]. In HCC, OV6 positive cells show a greater capability to form tumors resistant to conventional therapy, as well as metastatic potential and high invasiveness [64]. EpCAM-positive CSCs contribute to tumor establishment and invasiveness [9]. Thus, targeting these CSCs subsets has been an attractive strategy for HCC treatment. Our results showed that quercetin decreases the expression of OV6 and EpCAM markers induced by CT group. Quercetin has previously been shown to inhibit cell proliferation, induce cell cycle arrest, and the apoptosis of CD133-positive CSCs [65]. Furthermore, quercetin also suppresses the self-renewal capability and invasiveness of CSCs in breast cancer by regulating EpCAM expression [66]. Additionally, quercetin inhibits the activation of Wnt/β-catenin, a signaling pathway involved in liver CSCs establishment [67]. The Wnt/β-catenin signaling pathway regulates the expression of CXCR4, a central molecule in maintaining OV6-positive CSCs, which provides the capacities of self-renewal, stem cell-associated gene expression, tumorigenicity, and chemoresistance [68,69]. Our results show that quercetin decreases the level of OV6 and EpCAM protein, indicating that quercetin may be inhibiting the tumor establishment as early as in the appearance of preneoplastic lesions. Other authors have reported that quercetin inhibits the self-renewal capability of prostate CSCs, by inducing apoptosis and blocking the migration and invasion of CSCs [70]. Shen et al. have reported that quercetin inhibits the viability of gastric cancer stem cells by attenuating PI3K/AKT signaling pathway, and induces mitochondrial apoptosis pathway by reducing the mitochondrial membrane potential [71]. Thus, it is plausible to propose that in our HCC model quercetin might be also targeting this signaling pathway, which represents an attractive hypothesis that should be addressed.

Ideally, the effectiveness of therapeutic agents against HCC should be monitored through detection of specific biomarkers. Recently, ABCC3 transporter has been proposed as a marker of HCC progression. Under physiological conditions, ABCC3 is barely expressed in the liver, and its expression has been mainly reported in cholangiocytes and hepatocytes; ABCC3 has been observed in both basolateral membrane and cytoplasm [72]. However, ABCC3 is overexpressed in HCC, but mainly within the tumors and surrounding area [73,74]. Carrasco-Torres et al. have demonstrated that ABCC3 level increases as HCC develops in the MRHM, suggesting a direct relationship between ABCC3 expression and cancer progression [19]. In this line, elevated levels of ABCC3 have been observed in tissues from HCC patients [75]. Coincidentally, our results showed increased level of ABCC3 mainly in hepatocytes of preneoplastic lesions induced by CT group.

Drug resistance has been a huge challenge for anticancer therapies. Sorafenib is one of the first-line drugs for the treatment of HCC; however, its use generates drug resistance, which is associated with poor prognosis of treated patients [18]. One of the mechanisms involved in drug resistance acquisition is the overexpression of transporters of ABC family, which is closely associated with poor response to chemotherapy [17]; for example, ABCC3 is overexpressed in cells resistant to sorafenib treatment [18]. Our results show that quercetin reduces ABCC3 expression level, as well as the number and size of preneoplastic lesions. Of note, we also observed that quercetin does not increase the level of ABCC3 in NT + Q group; thus, quercetin has the capability to prevent drug resistance and as a result, to enhance the therapeutic effects of anticancer drugs. Cheng et al. have shown that quercetin reverses multidrug resistance by decreasing the expression of transporters ABCB1, ABCC1, and ABCC2 through the FZD/β-catenin pathway [76]. Furthermore, Ulasov et al. have shown that the combination of quercetin with doxorubicin increases the sensitivity of HCC cells to cancer treatment in part by decreasing the activity of the MDR1 transporter [77]. In this line, Wang et al. have demonstrated that the antioxidant tetramethylpyrazine reduces the expression of ABCC3 [78]; moreover, Zeng et al. have shown that the combination of curcumol with doxorubicin increases the sensitivity of tumors to anticancer treatment by decreasing the expression of ABCC3 in breast cancer [79]. Importantly, both in vitro and in vivo studies have demonstrated that quercetin exerts toxic activities selectively on transformed cells and has minimal effect on normal cells. For instance, Ji et al. have reported that quercetin inhibits the growth of HCC cells and has a lesser effect on the viability of normal hepatocytes [36], indicating that transformed cells have greater sensitivity to the cytotoxic effect of quercetin [80]. Additionally, it has been reported that quercetin does not alter liver histology in healthy rats when administered at low doses [32]. In this line, we also observed that quercetin did not affect the normal architecture of liver tissue in control animal but improved those subjected to carcinogenic treatment. Thus, our results support the notion that the use of flavonoids as a strategy to decrease drugs resistance could be effective, especially, in the early HCC stages.

## 5. Conclusions

Our results show that quercetin reduces the appearance of preneoplastic lesions and restructures the liver architecture. It also reduces the accumulation of glycogen, the level of myofibroblasts markers, and therefore fibrogenesis. In addition, quercetin also reduces CSCs subpopulations and drug resistance by decreasing ABCC3 levels. Therefore, it is plausible to propose the use of quercetin as an adjuvant agent to increase the therapeutic response in patients bearing HCC. It is important to highlight that our investigation is based on histological analyses; therefore, additional experiments are needed to elucidate the molecular mechanisms leading to preneoplastic lesions reversion in the rat liver. In conclusion, our results reveal that quercetin reverses the appearance of preneoplastic lesions by regulating some TME components and significantly reduces the acquisition of drug resistance in the early HCC stages.

## Data Availability

Data is contained within the article.

## Supplementary Materials

The following supporting information can be downloaded at: https://www.mdpi.com/article/10.3390/antiox11020358/s1, Table S1: Antibodies.
