# Supplementary material for: Quercetin Regulates Key Components of the Cellular Microenvironment during Early Hepatocarcinogenesis
Source: Antioxidants (Basel). 2022 Feb 11;11(2):358. doi: 10.3390/antiox11020358 (PMC8868318; doi:10.3390/antiox11020358)
Supplement: Supplementary file 1 [file antioxidants-11-00358-s001.zip › antioxidants-1553360-supplementary.pdf]

Table S1. Antibodys

Primary antibody	Epitope exposure	Primary antibody dilution	Secondary antibody	Secondary antibody dilution
OV6	Buffer Tris pH=10	1:30 in 3% BSA in TBS	Antimouse	1:200 in 1 % BSA in TBS
EpCam	Buffer Tris pH=10	1:100 in 3% BSA in TBS	Antimouse	1:150 in 3 % BSA in TBS
α-SMA	Buffer Citrates pH=6.0	1:700 in 5% BSA in PBS	Antimouse	1:300 in 5% BSA in PBS
ABCC3	Buffer Citrates pH=6.0	1:50 in 5% BSA in PBS	Antimouse	1:250 in 5% BSA in PBS
